# Does the extent of the surgical margin affect the likelihood of local recurrence in Patnaik grade I or II cutaneous mast cell tumours?

**DOI:** 10.18849/ve.v7i1.508

**Published:** 2022-03-10

**Authors:** Christos Dorlis+

**Keywords:** CANINE MAST CELL TUMOUR, CONSERVATIVE MARGINS, LOCAL RECURRENCE, SURGICAL MARGINS, WIDE MARGINS

## Abstract

PICO question

Does the extent of the surgical margin affect the likelihood of local recurrence in Patnaik grade I or II cutaneous mast cell tumours?

Clinical bottom line

Category of research question

Treatment

The number and type of study designs reviewed

Eight papers were critically reviewed. Five were retrospective case series, two prospective clinical trials, and one prospective case series

Strength of evidence

Low

Outcomes reported

Sequin et al. (2001) reported a local recurrence rate of 5%, but this study is 20 years old. In the studies of Simpson et al. (2004), Fulcher et al. (2006), Pratschke et al. (2013), Saunders et al. (2020), and Itoh et al. (2021), no local recurrence was observed in grade I and II mast cell tumours, while in the Milovancev et al. (2019) study, only 1/30 low-grade cutaneous mast cell tumors developed local recurrence. Therefore, there is some evidence that conservative surgical excision is sufficient to achieve local control with low recurrence rates

Conclusion

There is increasing evidence in the literature for conservative surgical excision of grade I and II MCTs, but because the quality of evidence is low, no clear recommendations can be made. Further studies are needed to determine recommendations for surgical excision of cutaneous MCTs based on the biological characteristics of the tumour and the completeness of histologic margins

## How to apply this evidence in practice

The application of evidence into practice should take into account multiple factors, not limited to: individual clinical expertise, patient’s circumstances and owners’ values, country, location or clinic where you work, the individual case in front of you, the availability of therapies and resources.

Knowledge Summaries are a resource to help reinforce or inform decision making. They do not override the responsibility or judgement of the practitioner to do what is best for the animal in their care.

## Clinical scenario

A 4 year old neutered male Pug presents with a 1 cm cutaneous mass in the left flank region. The owner reports that the mass has been there for 3 months and does not change in size. Cytologic examination suggests a mast cell tumour (MCT). Blood work and abdominal ultrasound were unremarkable. What margin would you ideally excise this mass?

## The evidence

The overall quality of evidence is low, consisting predominantly of retrospective observational studies (Sequin et al., 2001; Simpson et al., 2004; Pratschke et al., 2013; Chu et al., 2020; Saunders et al., 2020; and Itoh et al., 2021) and no randomised prospective clinical trials. Two prospective observational clinical studies (Fulcher et al., 2006; and Milovancev et al., 2019) showed a low recurrence rate, but both consisted of a very small sample size (23 and 30 cutaneous MCTs, respectively).

## Summary of the evidence

**Seguin et al. (2001):** 

Population:	Medical records of dogs with mast cell tumours (MCTs) were diagnosed at Colorado State University Veterinary Teaching Hospital (CSU-VTH) between January 1996 and August 1999. The study included only dogs with grade II (Patnaik) cutaneous MCTs treated with surgery alone. 21 of the included tumours were scars from previously incompletely excised MCTs.
Sample size:	55 dogs (60 MCTs).
Intervention details:	**Surgical margins**: 2–3 cm lateral margins and one fascial plane as deep margin. **Adjuvant treatment**: None. **Tumour grading**: Patnaik grading system. No Kiupel grading. **Histological margins**: were categorised as complete, complete but close (tumour cells within 1 mm of surgical margin), or incomplete (tumour cells at the surgical margin). **Follow-up method**: Follow-up information was obtained via reassessment at the clinic or telephone calls to referring veterinarians or owners.
Study design:	Retrospective case series.
Outcome studied:	Local recurrence. Completeness of excision. Time of local recurrence. Survival time (time from surgery until death).
Main Findings (relevant to PICO question)	**Tumour diameter**: Median tumour diameter (range): 2 cm (1–4 cm). Tumour grading: 39 grade II MCTs (Patnaik). 21 surgical scars from previously incompletely excised MCTs. **Tumour location**: The tumours were located on the head and neck = 8 (13%), trunk = 29 (48%), limb =18 (30%), genital region = 3 (5%) and location were not available for 2 (3%) tumours. **Histological margins results**: complete in 54 (90%) tumours, complete but close in 3 (5%) tumours, incomplete in 1 (2%) tumour, information not available in 2 (3%) tumours, two dogs had amputation for definite surgery, all 21 scar excision had complete histological margins. **Local recurrence**: three tumours (5%). **The median time to local recurrence** was 62 days (range, 51–252 days). **Median follow-up time **540 days (range, 77–1,804 days). 46/55 (84%) dogs were free of MCT during the study period.
Limitations:	Small sample size – risk of type II error. Two dogs had amputation for definite treatment and local recurrence was impossible to assess in these two cases. No clear definition of exact margins as a range was given (2–3 cm) and lacked assigned groups. Non-objective follow-up in some cases (client follow-up) – potential risk of local recurrence underestimated. Definition of local recurrence in relation to the original scar was not specified – the risk of some local recurrences being classified as de novo. Retrospective nature – some medical records are incomplete (e.g., tumour size) and therefore cannot provide information for tumours larger than 4 cm. No definite diagnosis of local recurrence with cytology or biopsy. Prognostic relationship between histopathological status of surgical margins and recurrence could not be determined because the number of local recurrences was small (5%), and the classification system of histological margins differed from other studies and was therefore difficult to compare. Participants’ characteristics not homogeneous – surgery was performed as revision in some cases (21 dogs).

**Simpson et al. (2004):** 

Population:	Client-owned dogs diagnosed with cutaneous mast cell tumours (MCTs) based on cytological examination of fine needle aspirates, at Animal Medical Center, New York, USA – time interval not reported.
Sample size:	21 dogs (23 cutaneous grade I and II (Patnaik) MCTs).
Intervention details:	**Surgical margins**: MCT removed with 3 cm margins and one fascial plane as deep margin. Prior to removal, the skin was marked at 1, 2, and 3 cm from the tumour margin at 0^°^, 90^°^, 180^°^, and 270^°^. Tumours were affixed to cardboard to recreate their original shape. Tissues were fixed in formalin and eight 1 cm long full-thickness tissue samples were taken at 3 cm margins. Four 1cm long full-thickness tissue sections were obtained at the 1cm and 2cm margins. All tissue margins were examined by one board-certified pathologist. **Adjuvant treatment**: One dog had radiation therapy after incomplete deep margin excision. **Tumour grading**: All tumours were graded by two pathologists using the Patnaik system. **Histological margins**: were categorised as complete, complete but close (tumour cells within 1 mm of surgical margin), or incomplete (tumour cells at the surgical margin). **Follow-up methods**: Follow-up information was obtained via reassessment at the clinic or telephone calls to referring veterinarians. **Local recurrence**: Disease-free interval was defined as the interval from the date of tumour excision to the date of local recurrence. **Survival time** was defined as the interval from the date of tumour excision to the date of death.
Study design:	Prospective case series.
Outcome studied:	Local recurrence. Survival time.
Main Findings (relevant to PICO question)	**Tumour diameter**: Mean tumour diameter was 1.9 cm (median, 1.7 cm; range, 0.35–5.0 cm). **Tumour location**: 15/23 (65%) tumours were located on the trunk, 5/23 (22%) were located on the hind limbs, and 3/23 (13%) were located on the neck. **Tumour grading**: There were three grade I and 20 grade II tumours. **Histological margins results**: All grade I tumours were completely excised at all margins. 15/20 (75%) of the grade II tumours were completely excised at the 1 cm margin. 20/20(100%) of grade II tumours were completely excised at the 2 cm margin. 2/20 grade II tumours had incomplete deep margins. **Local recurrence**: No local recurrence was noted in any dog. **Follow-up time**: The median follow-up time was 351 days (range, 78–603 days).
Limitations:	This study has a small sample size and is prone to type II error. This study does not include MCTs on the face or inguinal and perineal regions, and no recommendation can be made for these locations. One dog in which MCTs were detected within 1 mm of the deep margin received full-course radiation therapy; therefore direct comparison cannot be made for this patient. Although there is no statistical correlation between tumour diameter and completeness of excision, most tumours in this study were small. The period during which participants were enrolled in the study was not mentioned. Inconsistent method of follow-up.

**Fulcher et al. (2006):** 

Population:	Client-owned dogs with one or more cutaneous mast cell tumours (MCTs) diagnosed with a cytological examination of fine needle aspirates, at Animal Medical Center, New York, USA – time interval not reported.
Sample size:	16 dogs (23 cutaneous MCTs).
Intervention details:	**Surgical margins**: MCT removed with 3 cm margins and one fascial plane deep margin. Prior to removal, the skin was marked at 1, 2, and 3 cm from the tumour margin at 0^°^, 90^°^, 180^°^, and 270^°^. Tumours were affixed to cardboard to recreate their original shape. Tissues were fixed in formalin and eight 1 cm long full-thickness tissue samples were taken at 3 cm margins. Four 1cm long full-thickness tissue sections were obtained at the 1cm and 2cm margins. All tissue margins were examined by one board-certified pathologist. **Tumour grading:** All tumours were graded by two pathologists using the Patnaik system. **Adjuvant treatment: **Some dogs had corticosteroids, but no more information was given. **Histological margins** were categorised as complete, complete but close (tumour cells within 1 mm of surgical margin), or incomplete (tumour cells at the surgical margin). **Follow-up method**: Follow-up information was obtained via reassessment at the clinic or telephone calls to referring veterinarians. **Local recurrence** was defined as the development of an MCT at or within 2 cm of the original surgical site. **Disease-free interval:** time of surgery to local recurrence.
Study design:	Prospective clinical trial.
Outcome studied:	Local recurrence. Disease-free interval.
Main Findings (relevant to PICO question)	**Tumour diameter**: Mean tumour diameter was 1.3 cm (median, 1.1 cm; range, 0.4–3.1 cm). **Tumour location**: 10 (44%) located in the trunk, 7 (30%) on the hindlimbs, 3 (13%) on the forelimbs and 3 (13%) on the head and neck. **Tumour grading**: 4/23 (17%) MCTs were grade I tumours, 19/23 (83%) were grade II tumours. **Histological margins results**: All grade I tumours were completely excised at the 1- and 2cm margins. 13/19 grade II MCTs, no MCTs were detected at the 1cm margin. 17/19 tumours, no MCTs were detected at the 2cm margin. Overall, 21/23 (91%) MCTs were completely excised. There was no recurrence in two of the grade II tumours with incomplete margins, although one had revision surgery 2 weeks after the first excision. All had complete deep margins. **Local recurrence**: None of the dogs had local recurrence. **Follow-up time**: The median follow-up interval for all dogs was 379 days (range, 51–538 days).
Limitations:	This study has small sample size and is prone to type II error. This study does not include MCTs on the face or inguinal and perineal regions, and no recommendation can be made for these locations. One dog with incompletely excised MCT received additional surgery in 2 weeks therefore direct comparison cannot be made. Inconsistent method of follow-up (either clinical examination or telephone update). The period when participants were enrolled in the study was not mentioned.

**Pratschke et al. (2013):** 

Population:	Dogs with cutaneous and subcutaneous mast cell tumours (MCTs) were diagnosed either with cytological or histopathological examination at the University of Glasgow between 2008 and 2012.
Sample size:	40 dogs (47 MCT [41 cutaneous and six subcutaneous]).
Intervention details:	**Surgical margins**: Lateral margins equivalent to the widest measured diameter of the tumour and a minimum depth of one well-defined fascial plane. If tumour size exceeded 4 cm then a fixed 4 cm lateral margin was taken. **Tumour grading**: Grading based on Patnaik and Kiupel system. **Histological margins**: Margins were considered clear when the distance between neoplastic cells and non-neoplastic tissue was > 1 mm; otherwise, they were considered incomplete. **Follow-up methods**: retrieved from clinical records and through contact with the referring veterinarians and dog owners.
Study design:	Retrospective case series.
Outcome studied:	Retrospective case series.
Main Findings (relevant to PICO question)	**Tumour diameter**: Tumour diameter ranged from 5 mm to 60 X 40 mm. **Tumour location:** 24/47 (51%) located in the limbs, 9/47 (19%) on the trunk, 9/47 (19%) on the head, and 5 (11%) the inguinal region or prepuce and genital area. **Tumour grading**: 21 tumours grade I, 18 tumours grade II, two grade III, and six subcutaneous. **Adjuvant treatments**: Five low-grade MCTs (four cutaneous and one subcutaneous) had postoperative chemotherapy (all these five had nodal metastasis). One dog with incomplete margins had postoperative radiation therapy. Histological margins results: 40/47 (85%) tumours with complete and 7/47 tumors (15%) with incomplete margins. Of the excised tumours with incomplete margins, four had one incomplete lateral margin and three had an incomplete deep margin. 5/7 tumours with incomplete margins were located on the limbs. Of the dogs with incomplete excisional margins, one had postoperative radiation therapy (disease free interval [DFI]: 300 days) and one had chemotherapy (DFI: 510 days), one died from gastric dilatation-volvulus the day after surgery, one developed local recurrence and was euthanised 1.5 months later, and the other three had no adjunctive treatment and no identified recurrence at follow-up times of 120, 300, and 330 days. **Local recurrence**: Only one local recurrence (dog with grade III mast cell tumour completely excised). **Follow-up time**: Follow-up intervals ranged from 0 to 1,140 days after MCT excision (median, 420 days).
Limitations:	Retrospective nature – incomplete medical records may influence the outcome. Lack of control group for comparison (a group with 3 cm margins for example). Two dogs with incomplete margins have additional treatments and the other died from another cause and this may influence the low number of recurrences**.** Inconsistent follow-up methods (some cases had owner and referring veterinary surgeon follow-up and other reviewing the clinical records).

**Milovancev et al. (2019):** 

Population:	Client-owned dogs presented to the Veterinary Teaching Hospital at Oregon State University for surgical treatment of mast cell tumours (MCTs) between August 1, 2014–July 31, 2016. The population include: 38 dogs (52 MCTs), 50 low-grade (30 cutaneous and 20 subcutaneous), two MCTs high grade. MCTs diagnosed via cytological examination. The authors also looked at 19 soft tissue sarcoma cases, but they are not relevant to the PICO topic in this study, thus they will not be discussed further.
Sample size:	38 dogs (30 cutaneous low-grade MCTs).
Intervention details:	**Surgical margins**: en bloc removal of a grossly normal surgical margin of at least 1 mm beyond the palpable and visible edge of the tumour (i.e., no grossly visible tumour at the surgical margins). **Tumour grading**: based on Kiupel and Patnaik system. **Histological margins**: histological margins of 0 mm were considered as incomplete excision whereas histological margins >0 mm (i.e., tumour cells do not reach the inked surgical margin) were considered as complete excision, **Adjuvant treatment**: 4 low-grade tumours had additional chemotherapy (three were clinical stage III and one was clinical stage IV). **Follow-up methods**: 2 year postoperative active surveillance period with clinical outcome data. **Local recurrence **confirmed by cytological evaluation in a follow-up period of 24 months.
Study design:	Prospective clinical trial.
Outcome studied:	Local recurrence.
Main Findings (relevant to PICO question)	**Tumour diameter**: Median 15 mm, range 5–52 mm. **Tumour grading**: 30 low-grade MCTs (graded as II on Patnaik system and low-grade on Kiupel system). **Narrowest intraoperative margins**: Two MCTs: 5 mm One MCT: 8 mm Four MCTs: 10 mm Two MCTs: 15 mm 18 MCTs: 20 mm One MCT: 25 mm Two MCTs: 30 mm Narrowest histological margins: 0 mm: Four tumours <1 mm: 11 tumours Number of deep fascial planes: 0: Two tumours 1: 27 tumours 2: One tumour **1/30 low-grade MCT developed local recurrence**. This was a completely excised (narrowest histological margin = 10.6 m) cutaneous MCT from the ventral abdomen, with local recurrence noted at the 12 month postoperative recheck (confirmed via fine-needle-aspirate cytology). The dog was euthanised 1229 days postoperatively for progressive osteoarthritis.
Limitations:	Small number of cases – risk of type II error. No standardised margin of excision.

**Chu et al. (2020):** 

Population:	Cutaneous mast cell tumours (MCTs) excised from dogs between January 1, 2007, and December 31, 2017, at the Cornell University Hospital for Animals were identified from a search of the pathology department’s database. 623 cutaneous MCTs were identified (83 tumours grade I and II met the inclusion criteria).
Sample size:	68 dogs (83 tumours grade I and II [Patnaik grading system]).
Intervention details:	**Surgical margins**: Tumours were assigned to groups. Conservative-margin group (n = 46): if they had been excised with lateral margins that were 2 cm for tumours ≥ 2 cm in diameter or equal to the tumour diameter (proportional) for tumours < 2 cm in diameter. Wide-margin group (n = 37): if they had been excised with lateral margins of 3 cm, regardless of tumour diameter. A noninferiority margin of > 0.9 cm was used for the risk ratio (probability of complete excision for the conservative- vs wide-margin group). **Neoadjuvant treatment**: some dogs had preoperative prednisone administration. **Tumour grading system**: Patnaik and Kiupel grading system. Histological margins: Complete excision: classified when results of microscopic examination revealed no MCTs with characteristics suggestive of neoplastic behaviour at the lateral or deep edge of the section, whether individually scattered or as part of the neoplasm (Pratschke et al., 2013). Incomplete excision: all the other instances was classified as incomplete excision. **Follow-up method**: assessment of histological margins for completeness of excision.
Study design:	Retrospective observational study.
Outcome studied:	The margin of excision (complete vs incomplete excision as histologically determined) was compared between conservative- and wide-margin groups.
Main Findings (relevant to PICO question)	**Median (range) tumour diameter**: Wide-margin group: 1 cm (0.2–7.0 cm). Conservative-margin groups :1.0 cm (0.1–4.0 cm). **Tumour location**: limbs (29), thoracic region (25), abdominal region (11), head (five), and neck region (three). The study did not specify the location of the remaining 10 tumours. **Tumour grading**: All tumours were histologically classified as grade I or II by the Patnaik system. Tumours in the wide-margin group were more likely to be classified as grade II (vs grade I) than were tumours in the conservative-margin group. **Adjuvant treatments**: One dog in the wide margin group and two in the conservative margin group had preoperative prednisone administration. **Histological tumour-free margins**: Conservative group: 43/46 (93%). Wide margin group: 34/37 (92%). The risk ratio met the criterion for noninferiority.
Limitations:	Lack of randomisation: More tumours grade II in the wide margins group which could induce bias to the study. The outcome was assessed by histological complete / incomplete margins and there is no correlation with clinical recurrence – further studies are needed to assess these groups with clinical recurrence. Due to the retrospective nature 473 dogs were excluded due to missing information which may introduce selection bias. Histological margin assessment based on radial method only by different board-certified pathologists.

**Saunders et al. (2020):** 

Population:	Dogs presented to the Veterinary Oncology Specialists (Underwood, Australia) between July and October 2016 and Animal Referral Hospital Brisbane (Brisbane, Australia) between October 2016 and August 2018.
Sample size:	65 dogs (100 cutaneous mast cell tumours [MCTs]).
Intervention details:	**Surgical margins**: The width of proportional margin excision was based on the largest lateral diameter of the mass up to a level of 2 cm. If the tumour diameter was greater than 2 cm, the lateral margins that were used to excise the mass were limited to 2 cm. The tumours were excised in an en bloc manner with the proposed lateral surgical margins and a minimum of at least one fascial plane as deep margin. **Preoperative neoadjuvant medications**: prednisolone (0.5–1 mg/kg PO SID ± chlorpheniramine (0.5–1 mg/kg PO BID) for 7–10 days prior to surgical intervention. **Tumour grading**: all tumours graded with Kiupel system. **Tumour size**: Authors created two subgroups based on tumour size: Of the low-grade MCTs, 42 were < 10 mm in diameter, with the remaining 46 = 10 mm. Of the high-grade MCTs 5 were < 10 mm and seven were = 10 mm groups. Tissues were fixed in formalin and were reviewed by a board-certified pathologist. **Histological margins**: Complete excision of the mass was defined as both the lateral and deep margins being clear of infiltration with MCTs. Incomplete excision was defined as mast cell infiltration in lateral or deep margins. **Histological margin size** was grouped into three categories: ≤2 mm, 2.1–5 mm, and > 5 mm. **Follow-up method**: a follow-up period of at least 365 days by physical examination or owner-contact ± cytological evaluation when appropriate.
Study design:	Retrospective cohort study.
Outcome studied:	Local recurrence.
Main Findings (relevant to PICO question)	Tumour diameter: Low-grade MCTs: 42 were < 10 mm in diameter, and 46 being = 10 mm in diameter. High-grade MCTs: five were <10mm and seven were =10mm Median overall tumour diameter =10.0 mm (range of 1–85 mm). Median high-grade tumour diameter = 15.0 mm (range of 3–85 mm). Median low-grade tumours diameter = 10.0 mm (range of 1–45 mm). **Tumour location**: (35%) limbs, (23%) flank and abdomen, (15%) thorax, (15%) the prepuce and / or perineal regions, (12%) the head and neck. Tumour grading: 88 low-grade MCT and 12 high-grade MCT. 11 dogs had high grade MCTs (one had two tumours). 54 dogs had low-grade MCTs (18 multiple). **Histological margins/completeness of excision**: 95/100 (95%) MCTs were completely excised. Low-grade: 84/88 (96%). High grade:11/12 (92%). small tumours (< 10 mm): all had complete excision 47/47 (100%). large tumours (≥ 10 mm): 48/53 (91%) had complete. Histological tumour-free margins (HTFM) size: Median HTFM size of all complete excised tumours: 5 mm (range 0.5–15 mm). Median HTFM size of high-grade tumours: 5 mm (range 2–14 mm). Median HTFM size of low-grade tumours: 5 mm (range 0.5–15 mm). No significant association was evident between tumour grade and HTFM size. No significant association was evident between the tumour size groups and the HTFM size groups. **Recurrence rate**: 3/100 tumours (3%) with a median follow-up period of 593 days (range 180–1460 days) with none on the low-grade group and three on the high-grade group 3/12(25%). Of the incompletely excised masses, four were low-grade, and one was high-grade and only the high grade recurred. 45 tumours had neoadjuvant prednisolone and chlorpheniramine administered but there was no significant association between the rates of complete excision between tumours that had neoadjuvant therapy compared to those that did not.
Limitations:	Retrospective nature and as such inability to standardise follow-up assessment for recurrence. Some dogs had neoadjuvant prednisolone which could affect tumour size, completeness of excision, and margin assessment. 18 dogs were excluded from the margin assessment as no report on histological margins in the clinical records. Tumour size may affect the completeness of excision as this relationship was close to statistical significance – potentially with increased sample size groups, the relationship would be more obvious. Eight dogs lost to follow-up before the 365 days but all of them were grade I. Limitation is also the radial trimming of margins as less sensitive than the tangential method to detect residual tumours at the margins.

**Itoh et al. (2021):** 

Population:	Dogs with cutaneous and subcutaneous mast cell tumours (MCTs) presented to Aoba Animal Hospital between November 2013 and September 2018 and were treated with the proportional margin resection technique. 29 dogs had MCT surgery in this period. Six dogs were excluded because margins were wider or narrower than the proportional diameter. The final population was 23 dogs (25 MCTs). From the 25 MCTs, the 24 were cutaneous and one subcutaneous.
Sample size:	23 dogs (24 cutaneous MCTs).
Intervention details:	**Surgical margins**: MCTs excised with margins equal to the proportional diameter of the tumour as described by Pratschke et al. (2013) and at least one fascial plane as deep margin. **Tumour grading**: All tumours graded based on Patnaik and Kiupel system. **Adjuvant treatment**: No dogs had adjuvant treatment. **Histological margins**: Classified as complete, close (tumour cells within 1 mm of the margin), or incomplete (tumour cells in the margin). **Follow-up method:** Review of the medical records.
Study design:	Retrospective case series.
Outcome studied:	Local recurrence. Completeness of resection.
Main Findings (relevant to PICO question)	**Tumour diameter**: The tumour diameter range was 0.3–2.6 cm (Median: 0.9 cm). **Tumour location**: Trunk (12), extremities (seven), head (two), vagina / perineum (three), and tail (one). **Tumour grading**: All 24 cutaneous MCTs grades as low-grade (Kiupel). Five MCTs Patnaik grade I and 19 as Patnaik grade II. Histological margins results: Complete in 20 cases. Close (deep margin) in two cases. Incomplete (deep margin) in two cases. **Local recurrence**: No local recurrence in a follow-up period of 410–2,219 days (median: 990 days).
Limitations:	Local recurrence distance in relation to surgical scar was not specified. Small sample size – prone to type II error. Only tumours with small size were included.

## Appraisal, application and reflection

Historically, it has been recommended that mast cell tumours (MCTs) independently of grade, must be excised with 3 cm lateral margins and one fascial plane as deep margin. However, evidence to support this recommendation is lacking, especially for grade I and II MCTs. Recent literature suggests that the removal of these low-grade tumours with 2 cm lateral margins and one deep fascial plane, is sufficient to achieve local control and does not increase the incidence of local recurrence (Simpson et al., 2004; Fulcher et al., 2006; Chu et al., 2020; Milovancev et al., 2019; and Saunders et al., 2020).

In the study by Seguin et al. (2001), 2–3 cm margins were used to remove MCTs, however, the authors did not divide the dogs into groups in terms of margins, so no direct comparison between conservative and wide excision is possible. In this study, only 5% (three tumours) recurred locally; however, the authors based their follow-up not only on clinical examinations but also on referring veterinarians or telephone updates from clients. There is a possibility that this local recurrence rate is underestimated, as some recurrences may be missed by clients. The authors also included dogs with scar tissue of previously incompletely excised MCTs, which makes the study population not homogeneous. In addition, two dogs had an amputation as definite treatment, and as such local recurrence could not be evaluated in these dogs. Other limitations of this study include its retrospective nature, which may mean that some information was not recorded in the medical records.

Two later studies, one by Simpson et al. (2004) and Fulcher et al. (2006), investigated the removal of grade I and II MCTs with 2 cm lateral margins. Both studies suffer from small sample sizes, which predispose them to type II statistical error. When we combine both studies for a total number of 46 MCTs (seven grades I and 39 grade II), 44/46 MCTs (95%) were completely excised at 2 cm lateral surgical margin. Only two dogs, in the Fulcher et al. (2006) study, had incomplete histological margins and none of them suffered local recurrence; however, one dog received revision surgery 2 weeks later. In both studies, no local recurrence was noted at a median follow-up of 351 days and 379 days, respectively. Based on these studies, it was suggested that a surgical dose of 2 cm lateral margins and one fascial plane deep should be sufficient to achieve local control without increased rates of local recurrence for grade I and II MCTs.

Pratschke et al. (2013) first described the approach of surgical excision based on the proportional margin of the tumour. In this study, masses were resected with a lateral margin equal to their widest diameter, with a maximum lateral margin of 4 cm and a well-defined deep fascial plane. Complete excision was achieved in 40/47 (85%) of cases, and although 7/47 (15%) of excised tumours had dirty margins, only one recurred locally during a median follow-up of 420 days. This was a completely excised, grade III MCT, highlighting the fact that achieving local control is not only influenced by the completeness of histological margins but also depends on the biological behaviour of the tumour. This study had a small sample size and a relatively short median follow-up time, which may have influenced the local recurrence rate. Furthermore, two of the dogs with incomplete margins had adjuvant treatments and another dog with incomplete margins died from gastric dilatation-volvulus 1 day post-surgery. One advantage of this study is that it included tumours in difficult locations such as the face, distal extremities, inguinal and preputial regions.

Based on the original work by Pratchke et al. (2013), two recent studies used a modified proportional margins approach for MCT excision. Chu et al. (2020) compared the completeness of excision (histologically free of tumour margins) after conservative surgical excision (excision with lateral margins equal to or smaller than 2 cm) and wide surgical excision (3 cm lateral margins) and showed a similar percentage of histologically free margins in both groups (93% and 92%, respectively). The authors concluded that this conservative approach was not inferior to the wide approach in achieving histologically free margins. The main limitation of this study was that the outcome measured was histologically free margins and not local recurrence. Milovancev et al. (2019) showed in a prospective clinical study with a follow-up of 2 years that narrow histological margins (1 mm) do not always correlate with local recurrence. In this study, 30 low-grade MCTs with a mean intraoperative margin of 20 mm were removed and only one recurred.

Saunders et al. (2020) used a similar approach to Pratchke et al. (2013) and Chu et al. (2020), where they excised MCTs based on the largest lateral diameter of the mass up to 2 cm and with 2 cm margins for masses greater than 2 cm. This study contains the largest sample size with 100 MCTs but both low-grade (88) and high-grade (12) tumours were included. The local recurrence rate was 3% (3/100), similar to Pratchke et al’s. (2013) study 1/45 (2%) but occurred only in high-grade tumours. None of the low-grade mast cell tumours included in the study recurred locally.

A correlation between conservative versus wide surgical excision and completeness of histological margins could not be established in this review because the classification methods of histologic margins were inconsistent between studies and the sampling methods used by histopathologists were different. In addition, the clinical significance of surgical margins and completeness of excision with the likelihood of metastatic spread could not be evaluated in this review.

Increased tumour size is a significant risk factor for incomplete excision with a 1.4% increase in the risk of incomplete excision per cm^2^ (Monteiro et al., 2011). The studies in this review failed to show a significant association between tumour diameter and completeness of excision and clear recommendations regarding surgical margins and large diameter tumours (>4 cm) cannot be made because most of the tumours in the included studies were small in diameter.

MCTs in muzzle, prepuce, inguinal and perineal areas have been associated with aggressive behaviour (Hillman et al., 2010; and Sfiligoi et al., 2005). In Simpson et al. (2004), the location of the MCT on the hind limb was significantly associated with incomplete excision and in Pratchke et al’s. (2013) study, 5/7 tumours with incomplete margins were located on the limbs. This review cannot evaluate surgical margins and recurrence rates in MCTs in difficult locations such as the muzzle, distal extremities, inguinal and perineal areas due to the small number of these tumours in the study populations.

This review suggests that most grade I and II cutaneous MCTs can be safely excised without an increase in the recurrence rate using either 2 cm lateral margins (Simpson et al., 2004; and Fulcher et al., 2006) or the proportional margin approach (Pratschke et al., 2013; Chu et al., 2020; and Saunders et al., 2020) instead of the classic recommendation of wide surgical excision (3 cm lateral margins). Nevertheless, the overall evidence is low and further studies, preferably multi-institutional studies with larger study populations, are needed to provide higher quality of evidence. These recommendations do not apply to high-grade MCTs, which are biologically more aggressive, locally invasive, and more likely to metastasize. Similarly, these recommendations must be used with caution for subcutaneous MCTs. There is no grading system for subcutaneous MCTs and many times their actual margins are not easily distinguished due to their subcutaneous origin and surrounding oedema. Studies by Pratchke et al. (2013) and Itoh et al. (2021) contained subcutaneous MCTs, but due to their low numbers, no correlation between surgical margins and local recurrence could be made.

## Methodology Section

**Search Strategy:** 

Databases searched and dates covered:	CAB Abstracts accessed via OVID platform (1980–2021) PubMed database accessed via NCBI (1986–November 2021)
Search strategy:	CAB Abstracts: (Dog OR canine OR canines OR bitch OR canid) AND (mast cell tumour OR mast cell tumor) AND (surgical margin OR incomplete margin OR dirty margin OR surgical resection OR surgical excision OR surgery OR margin OR resection) AND (recurrence OR local control OR neoplasm recurrence) PubMed: (Dog OR canine OR canines) AND (mast cell tumour OR mast cell tumor) AND (surgical margin OR incomplete margin OR dirty margin OR surgical resection OR surgical excision OR surgery) AND (recurrence OR local control OR neoplasm recurrence)
Dates searches performed:	09 Nov 2021

**Exclusion / Inclusion Criteria:** 

Exclusion:	Single case reports, duplicate articles, review articles, book chapters, or sections. Articles which did not directly evaluate the relationship between lateral surgical margins and local recurrence. Articles that did not specify the surgical margins at the time of the surgery. Articles not available in the English language.
Inclusion:	Original peer-reviewed articles including case series, observational, or interventional studies which evaluate the relationship of lateral surgical margins in MCT excision and local recurrence**.**

**Search Outcome:** 

Database	Number of results	Excluded – Not relevant to PICO question	Excluded – Surgical margins at the time of the surgery not reported and studies with only subcutaneous MCTs	Excluded – Reviews, case reports and letters	Excluded – Not in English or not available	Total relevant papers
CAB Abstracts	31	9	5	10	1	6
PubMed	99	58	14	19	0	8
Total relevant papers when duplicates removed						8

## Conflict of Interest

The author declares no conflicts of interest.

## Disclaimer

Knowledge Summaries are a peer-reviewed article type which aims to answer a clinical question based on the best available current evidence. It does not override the responsibility of the practitioner. Informed decisions should be made by considering such factors as individual clinical expertise and judgement along with patient’s circumstances and owners’ values. Knowledge Summaries are a resource to help inform and any opinions expressed within the Knowledge Summaries are the author’s own and do not necessarily reflect the view of the RCVS Knowledge. Authors are responsible for the accuracy of the content. While the Editor and Publisher believe that all content herein are in accord with current recommendations and practice at the time of publication, they accept no legal responsibility for any errors or omissions, and make no warranty, express or implied, with respect to material contained within. For further information please refer to our Terms of Use.
